# Supplementary material for: Protocol: Weight-adjusted effective volume of 0.5% ropivacaine for combined costoclavicular brachial plexus block–cervical plexus blocks undergoing arthroscopic shoulder surgery: A dose-finding study protocol
Source: PLoS One. 2025 May 16;20(5):e0324135. doi: 10.1371/journal.pone.0324135 (PMC12083839; doi:10.1371/journal.pone.0324135)
Supplement: S1 Chinese Protocol — (DOCX) [file pone.0324135.s002.docx]

研究方案

（基于体重的0.5%罗哌卡因在肋锁间隙联合颈丛神经阻滞用于肩关节镜手术的量效研究）

Weight-adjusted effective volume of 0.5% ropivacaine for costoclavicular-cervical plexus blocks undergoing arthroscopic shoulder surgery: a dose-finding study

#### 研究背景

肩袖损伤在临床上非常常见，尤其在老年患者中。[1]目前，肩关节镜下肩袖修补术在临床上应用越来越广泛。[2]肌间沟臂丛神经阻滞（ISB）是控制肩关节术后疼痛的经典技术，相比单纯的全身麻醉，它可以显著缩短住院时间，降低术后疼痛评分。[3]然而，隔神经阻滞率较高。[4]

近期，有研究发现在肩关节镜手术中，肋锁间隙联合颈丛神经阻滞(CCB-CPBs)也能产生和ISB类似的镇痛效果。另外，CCB-CPBs的HDP发生率低于ISB，因此它可能是一种更安全的镇痛方式。[5,6]随后，一项尸体解剖研究发现，CCB可以阻滞腋神经和肩胛上神经，这两条神经是支配肩关节感觉的主要神经分支。[7]

因此，目前证据显示CCB-CPBs可以为肩关节镜手术提供术后镇痛，然而，过大的局麻药容量仍然可能引起HDP[8]；过小的局麻药容量可能引起镇痛不足。因此，探索提供完善术后镇痛效果同时避免HDP的CCB-CPBs最佳剂量是目前临床上急需解决的问题。

1. **、研究目的**

本研究通过确定CCB-CPBs用于肩关节镜手术全麻术后镇痛所需罗哌卡因 50%有效剂量（ED50）和 95%有效剂量（ED95），探索CCB患者肩关节镜手术全麻术后镇痛所需罗哌卡因的最佳剂量。

1. **、研究设计**

**3.1整体的研究设计和计划**

**3.2研究人群**

本研究计划纳入在全麻复合CCB-CPBs麻醉下进行肩关节镜手术的患者。

**3.2.1入选标准**

美国麻醉医师学会（ASA）Ⅰ～Ⅱ级

年龄18～75岁

体重指数在18～30kg/m^2^

**3.2.2排除标准**

对酰胺类局麻药过敏

患肢神经损伤或者感觉异常

锁骨下窝手术史

有出血倾向或者凝血功能障碍

穿刺部位感染

精神、语言或听力障碍

**3.3.3病例数及分组方法**

肋锁间隙联合颈丛神经阻滞(CCB-CPBs)，病例数：40例

1. **、研究步骤**

**4.1麻醉前准备**

术前访视产妇，记录患者的一般情况，包括年龄、身高、体重、既往史、手术史、过敏史。完善术前检查，排除麻醉和手术禁忌。术前常规禁食 6-8小时，禁饮 2 小时，无任何术前用药。

患者入室后，研究者详细介绍本研究的方案和意义。患者同意后，签署知情同意书，开放上肢静脉，给予10 ml/kg乳酸林格氏液扩容。常规监测无创血压（NIBP）、心电图（ECG）和脉搏血氧饱和度（SPO_2_）。

**4.2超声引导下肋锁间隙联合颈丛神经阻滞**

患者平卧于水平的手术床，手术侧肩部下垫软垫。使用高频线阵超声探头，探头平行放置在患侧锁骨下缘。患者头转向非手术侧，患肢可以适度地外展，用来获得最佳的锁骨下肋锁间隙视图。理想的超声视图是臂丛神经三束依次排列在腋动脉外侧，然而，考虑到患者因疼痛无法移动患肢，因此不强迫患者移动患肢。麻醉实施者调整超声探头方向和位置，获得最佳的视图后，消毒拟穿刺部位，采用严格的无菌措施实施后续的操作。使用30ml注射器，注射器内包含麻醉前配制的0.5%的罗哌卡因30ml。穿刺点使用1%利多卡因2-3ml进行皮肤浸润麻醉。使用平面内技术，神经刺激针从外侧到内侧，后束注射等剂量的局麻药。

所有患者接受超声引导下颈浅丛神经阻滞补充肩部的镇痛，使用0.5%罗哌卡因10ml左右。

**4.3基于体重的局麻药剂量分配**

我们设置初始容量为0.45ml/kg。根据上下序贯法，容量通过0.05ml/kg的增量或减量变化，由前一个受试者的反应决定。如果前一个受试者的术后疼痛评分≤3分，则定义为阻滞成功，下一个受试者的容量减少。如果前一个受试者的术后疼痛评分＞3分，则定义为阻滞失败，下一个受试者容量增加。

**4.3全麻管理**

所有患者均接受喉罩插管全身麻醉，全麻诱导使用丙泊酚（2-3mg/kg），根据患者心率，酌情使用阿托品（0.25-0.5mg），麻醉维持使用七氟烷（1.5-3%）。当心率或血压超过术前值的20%时，静脉推注芬太尼（50µg），允许多次使用。手术结束时，静脉推注托烷司琼5mg预防恶心呕吐。

术后所有患者接受静脉术后自控镇痛泵（PCIA）, 内含托烷司琼10mg和布托啡诺6mg。

**4.4观察指标**

**4.4.1主要指标**

离开手术室前的疼痛评分

**4.4.2次要指标**

1) 术中芬太尼消耗量

2) 神经阻滞的实施时间

3) 神经阻滞不良反应（刺破血管、感觉异常、声音嘶哑、霍纳综合征和局麻药中毒）

4) 阿片类不良反应（术后恶心呕吐或瘙痒）

5) 使用超声M模式评估膈肌移动度。患者在平卧位时，将凸陈探头放置在腋前线或锁骨中线和肋缘交界处，探头方向指向头侧，调整探头找到膈肌移动度最大位置,使用M模式,采样线与膈肌线垂直,取3个较稳定波形冻结分别测量每个波形移动度并取平均值。在麻醉前和离开恢复室前测量平静呼吸和深呼吸时的膈肌移动度。麻痹定义为深呼吸时，相比麻醉前，膈肌移动度降低≤25%为膈肌功能正常，25%<膈肌移动度降低<75%为膈肌部分麻痹，膈肌移动度降低≥75%、膈肌无运动或矛盾运动为膈肌完全麻痹

6) 术后一周，随访患者的满意度和神经阻滞并发症（持续麻木、感觉异常或者活动障碍）

1. **、统计分析**

采用SPSS 25.00统计软件进行统计分析。Kolmogorov-Smirnov检验用于检验数据分布的正态性。符合正态分布数据采用独立样本t检验分析，计量资料以平均值±标准差（SD）表示。正态分布数据采用独立样本t检验分析，非正态分布数据采用Mann-Whitney U检验进行评估，计量资料以中位数（区间）表示。计数数据采用卡方检验或Fisher精确检验进行分析。P<0.05的值被认为具有统计学意义。使用上下序贯法来估计患者的ED50和ED95。

1. **、研究相关伦理学**

**6.1伦理委员会的审核**

**本研究方案及与患者相关的资料必须提交伦理委员会的审核，获得伦理委员会书面同意之后方可开展。**

1. **、保密措施 本研究的结果可能会在医学杂志上发表，但是我们会按照法律的要求为患者的信息保密，患者的个人信息不会被泄露。必要时，政府管理部门和医院伦理委员会及其有关人员可以按规定查阅患者的资料。**
2. **、研究的预期进度和完成日期**

本研究预期2024年10月启动，实施时间为1年，2025年10月完成。

#### 参考文献

1. Teunis T, Lubberts B, Reilly BT, et al. A systematic review and pooled analysis of the prevalence of rotator cuff disease with increasing age. Journal of shoulder and elbow surgery. 2014 Dec;23(12):1913-1921.

2. Karjalainen TV, Jain NB, Heikkinen J, et al. Surgery for rotator cuff tears. Cochrane Database Syst Rev. 2019 Dec 9;12(12):Cd013502.

3. Yan S, Zhao Y, Zhang H. Efficacy and safety of interscalene block combined with general anesthesia for arthroscopic shoulder surgery: A meta-analysis. J Clin Anesth. 2018 Jun;47:74-79.

4. Tran DQ, Layera S, Bravo D, et al. Diaphragm-sparing nerve blocks for shoulder surgery, revisited. Reg Anesth Pain Med. 2019 Sep 20.

5. Jo Y, Oh C, Lee WY, et al. Randomised comparison between superior trunk and costoclavicular blocks for arthroscopic shoulder surgery: A noninferiority study. Eur J Anaesthesiol. 2022 Oct 1;39(10):810-817.

6. Aliste J, Bravo D, Layera S, et al. Randomized comparison between interscalene and costoclavicular blocks for arthroscopic shoulder surgery. Reg Anesth Pain Med. 2019 Jan 11.

7. Koyyalamudi V, Langley NR, Harbell MW, et al. Evaluating the spread of costoclavicular brachial plexus block: an anatomical study. Reg Anesth Pain Med. 2020/10/08 ed2021. p. 31-34.

8. Sivashanmugam T, Maurya I, Kumar N, et al. Ipsilateral hemidiaphragmatic paresis after a supraclavicular and costoclavicular brachial plexus block: A randomised observer blinded study. Eur J Anaesthesiol. 2019 Oct;36(10):787-795.
