# Supplementary material for: Protocol: Weight-adjusted effective volume of 0.5% ropivacaine for combined costoclavicular brachial plexus block–cervical plexus blocks undergoing arthroscopic shoulder surgery: A dose-finding study protocol
Source: PLoS One. 2025 May 16;20(5):e0324135. doi: 10.1371/journal.pone.0324135 (PMC12083839; doi:10.1371/journal.pone.0324135)
Supplement: S2 English Protocol — (DOCX) [file pone.0324135.s003.docx]

Research protocols

### Weight-adjusted effective volume of 0.5% ropivacaine for costoclavicular-cervical plexus blocks undergoing arthroscopic shoulder surgery: a dose-finding study

#### INTRODUCTION:

Rotator cuff injuries are very common clinically, especially in older patients.[1] Presently, Shoulder arthroscopic rotator cuff repair is becoming more and more widely used in clinical practice.[2] Interscalene brachial plexus block（ISB）It is a classic technique to control pain after shoulder surgery. Compared with general anaesthesia alone, it can significantly shorten the length of hospital stay and reduce postoperative pain scores.[3] However, the rate of septal nerve block is high.[4]

Recent It has been found that costoclavicular space combined with cervical plexus block (CCB-CPBs) can also produce similar analgesic effects as ISB in shoulder arthroscopic surgery. In addition, CCB-CPBs have a lower incidence of HDP than ISB, so it may be a safer form of analgesia.[5,6] Subsequently, an autopsy study found that CCB could block the axillary nerve and suprascapular nerve, which are the main nerve branches innervating sensation in the shoulder joint. [7]

Thus, current evidence suggests that CCB-CPBs can provide postoperative analgesia for shoulder arthroscopic surgery, however, excessive local anesthetic volume may still cause HDP [8]; A small local anesthetic volume may cause inadequate analgesia. Therefore, exploring the optimal dose of CCB-CPBs to provide a perfect postoperative analgesic effect while avoiding HDP is an urgent clinical problem.

#### OBJECTIVE

This study explored the optimal dose of ropivacaine for analgesia after shoulder arthroscopic surgery by determining the 50% effective dose (ED50) and 95% effective dose (ED95) of ropivacaine required for postoperative analgesia after shoulder arthroscopic surgery by CCB-CPBs for analgesia after general anesthesia for shoulder arthroscopic surgery.

#### METHODS

##### Subjects

This study plans to include 40 patients who underwent shoulder arthroscopic surgery under anesthesia under general anesthesia combined with CCB-CPBs.

##### Inclusion Criteria

American College of Anesthesiologists (ASA) Class I~II

Age 18~75 years old

The body mass index is 18~30kg/m^2^

##### Exclusion Criteria:

Allergy to amide local anesthetics

Nerve damage or paresthesias in the affected limb

History of subclavian fossa surgery

Have bleeding tendencies or coagulopathy

Infection at the puncture site

Mental, speech, or hearing impairments

##### Study Protocol

No premedication will be given to the patients, and they will be asked to observe an eight-hour fasting period. Once they enter the operating room, we will establish a peripheral intravenous access in the non-operative upper limb using a 16-gauge catheter for fluid administration. A rapid peripheral intravenous preload using Ringer's lactate solution will be commenced at a rate of 10 ml/kg, and this rate will be sustained throughout the entire procedure. Standard non-invasive monitoring methodologies, including non-invasive blood pressure measurement, pulse oximetry, and electrocardiography, will be implemented.

###### Baseline diaphragmatic excursion measurement

The patient will be positioned in a semi-sitting position with the head raised approximately 30 degrees. Via the anterior subcostal route, either the liver or the spleen will serve as an acoustic window. The excursion of the hemidiaphragm will be measured by utilizing the M-mode which will be equipped with a low-frequency (1 to 5 MHz) ultrasound probe. Stable waveforms will be documented during deep breathing, and the corresponding values will be recorded.

###### Ultrasound-guided costoclavicular space combined with cervical plexus block

The patient lies flat on a horizontal operating table with a soft cushion under the shoulder on the surgical side. A high-frequency linear array ultrasound probe is used, which is placed parallel to the inferior border of the clavicle on the affected side. The patient's head is turned to the non-surgical side, and the affected limb can be moderately abducted to obtain the best view of the subclavian costoclavicular space. The ideal ultrasound view is that the three bundles of the brachial plexus are arranged sequentially on the lateral aspect of the axillary artery, however, the patient is not forced to move the affected limb given that the patient is unable to move the affected limb due to pain. After adjusting the direction and position of the ultrasound probe to obtain the best view, the anesthesia practitioner disinfects the proposed puncture site and implements the subsequent operation with strict aseptic measures. Use a 30 ml syringe containing 30 ml of 0.5% ropivacaine prepared before anesthesia. The puncture point is anesthetized with 2-3 ml of 1% lidocaine for skin infiltration. Using the in-plane technique, the nerve stimulation needle is injected from lateral to medial, posterior bundle injection of an equal dose of local anesthetic.

All patients received ultrasound-guided superficial cervical plexus block to supplement shoulder analgesia with about 10ml of 0.5% ropivacaine.

###### Local anesthetic dose allocation based on body weight

We set the initial capacity to 0.45ml/kg. According to the upper and lower sequential method, the volume varies by 0.05 ml/kg in increments or decreases, determined by the response of the previous subject. If the postoperative pain score of the previous subject ≤ 3 points, it is defined as a successful block and volume reduction in the next subject. If the postoperative pain score of the previous subject is > 3 points, it is defined as block failure and volume increase for the next subject.

###### General anesthesia management

All patients received general anesthesia with laryngeal mask intubation, propofol (2-3 mg/kg) induced under general anesthesia, atropine (0.25-0.5 mg) as appropriate, and sevoflurane (1.5-3%) for anesthesia maintenance according to the patient's heart rate. When the heart rate or blood pressure exceeds 20% of the preoperative value, an intravenous bolus of fentanyl (50 μg) is allowed, multiple uses are allowed. At the end of the procedure, an intravenous bolus of tropisetron 5 mg is given to prevent nausea and vomiting.

All patients received intravenous postoperative self-controlled analgesic pump (PCIA) containing tropisetron 10 mg and butorphanol 6 mg.

#### Measurements

The primary outcome is the patients' NRS score before leaving the PACU.

The secondary outcomes are as follows:

1. The ipsilateral diaphragmatic excursion.

2. The occurrence of complications, and the consumption of fentanyl.

### Statistical analysis

SPSS 25.00 statistical software was used for statistical analysis. The Kolmogorov-Smirnov test is used to test the normality of the data distribution. The normally distributed data were analyzed using the independent samples t-test, and the continuous data were expressed as mean ± standard deviation (SD). The independent samples t-test was used for the normal distribution data, and the Mann-Whitney U test was used for the evaluation of the non-normal distribution data, and the continuous data were expressed as median (intervals). Count data were analyzed using either Chi-square test or Fisher's exact test. A value of P<0.05 was considered statistically significant. The upper and lower sequential methods were used to estimate the patient's ED50 and ED95.

### Research related ethics

#### Review by the Ethics Committee

The study protocol and patient-related information must be submitted to the Ethics Committee for review and written consent from the Ethics Committee before it can be carried out. Confidentiality The results of this study may be published in a medical journal, but we will keep the patient's information confidential as required by law, and the patient's personal information will not be disclosed. When necessary, government management departments, hospital ethics committees and their relevant personnel may consult patients' information in accordance with regulations.

#### Expected progress and completion date of the study

The study is expected to start in October 2024, with an implementation time of one year and completion in October 2025.

### References

1. Teunis T, Lubberts B, Reilly BT, et al. A systematic review and pooled analysis of the prevalence of rotator cuff disease with increasing age. Journal of shoulder and elbow surgery. 2014 Dec;23(12):1913-1921.

2. Karjalainen TV, Jain NB, Heikkinen J, et al. Surgery for rotator cuff tears. Cochrane Database Syst Rev. 2019 Dec 9;12(12):Cd013502.

3. Yan S, Zhao Y, Zhang H. Efficacy and safety of interscalene block combined with general anesthesia for arthroscopic shoulder surgery: A meta-analysis. J Clin Anesth. 2018 Jun;47:74-79.

4. Tran DQ, Layera S, Bravo D, et al. Diaphragm-sparing nerve blocks for shoulder surgery, revisited. Reg Anesth Pain Med. 2019 Sep 20.

5. Jo Y, Oh C, Lee WY, et al. Randomised comparison between superior trunk and costoclavicular blocks for arthroscopic shoulder surgery: A noninferiority study. Eur J Anaesthesiol. 2022 Oct 1;39(10):810-817.

6. Aliste J, Bravo D, Layera S, et al. Randomized comparison between interscalene and costoclavicular blocks for arthroscopic shoulder surgery. Reg Anesth Pain Med. 2019 Jan 11.

7. Koyyalamudi V, Langley NR, Harbell MW, et al. Evaluating the spread of costoclavicular brachial plexus block: an anatomical study. Reg Anesth Pain Med. 2020/10/08 ed2021. p. 31-34.

8. Sivashanmugam T, Maurya I, Kumar N, et al. Ipsilateral hemidiaphragmatic paresis after a supraclavicular and costoclavicular brachial plexus block: A randomised observer blinded study. Eur J Anaesthesiol. 2019 Oct;36(10):787-795.
